# Editorial: Oncoplastic surgery for breast cancer

**DOI:** 10.3389/fonc.2023.1348964

**Published:** 2024-03-27

**Authors:** René Aloisio da Costa Vieira, Gil Facina, Daniel Guimarães Tiezzi, Cicero de Andrade Urban, Ruffo de Freitas Junior

**Affiliations:** ^1^ Barretos Cancer Hospital, Barretos, Brazil; ^2^ Medical School of São Paulo State University, Botucatu, Brazil; ^3^ Federal University of São Paulo, São Paulo, Brazil; ^4^ University of São Paulo, Ribeirão Preto, Brazil; ^5^ Hospital Nossa Senhora das Graças, Curitiba, Brazil; ^6^ Faculty of Medicine, Federal University of Goiás, Goiânia, Brazil

**Keywords:** surgical flaps, breast conserving surgeries, breast neoplasms, breast reconstruction, oncoplastic surgery, extreme oncoplasty, oncoplastic reconstruction

The surgical treatment of breast cancer has changed radically. We have moved from radical mastectomy to conservative treatment of the breast and axilla, as it was observed that less radical treatment did not change survival. Our concept of surgical margins has also changed, and we have begun to better understand tumour biology through molecular subtypes (1). We have begun to evaluate local recurrence, reduction of sequelae, cosmesis and quality of life.

In the 1990s, Audrestch proposed the term oncoplasty (2), and this concept has evolved. Regarding breast conservative treatment (BCT), non-oncological mammoplasty opened space for a set of techniques to be used in the treatment of breast cancer. The benefits of aesthetic techniques were assimilated, leading to improvements in breast cosmesis and quality of life. Such techniques were improved and adjusted according to the location of the tumor and the resection volume. Aiming at dissemination, standardization and medical education, classifications emerged. Initially, Urban C (2008) (3) proposed three levels of competence. Clough K.B. et all (2010) (4), proposed a classification based on the resected volume, and finally, the American Society of Breast Surgeons (ASBS, 2019) (5, 6) defined the term volume displacement for redistributing the resection volume. They also considered the term volume replacement, in the context of implant-based reconstruction or local/regional flap reconstruction (5).

Radical mastectomy, which was initially associated with delayed reconstruction with myocutaneous flaps, was modified with immediate breast reconstruction techniques that allow preservation of the skin and nipple with the addition of breast prostheses. Aiming to improve the results, we started using dermal matrices, subcutaneous prostheses and fat grafts. In some centres, robotic and video-assisted mastectomy are already performed. The term oncoplastic surgery has been associated with BCT, but some non-American authors associate it with breast reconstruction.

Although axillary surgery has decreased, the spectrum of breast surgeries has expanded, making it necessary to have knowledge and training in the range of surgical possibilities. Neoadjuvant chemotherapy allows the initial reduction of tumors, and resection of residual disease is increasing the rate of BCT (1). The concept of tumor size has shifted to a breast/volume ratio. Then, the concept of extreme oncoplasty emerged (2015) (7), allowing BCT for tumors larger than 5 cm, with multifocal and/or multicentric disease. Oncoplasty represents a new paradigm to be disseminated and assimilated by breast surgeons (8).

When evaluating publications related to breast oncoplasty in PubMed (oncoplastic surgery or oncoplasty), we began to observe an increasing annual publication rate, summing up to a total number of 1,415 references in 2022. These publications cover four main topics (9): (1) indication: type of patient and tumor; (2) type of surgery: technique, oncological safety, laterality, symmetrization, and local recurrence-free survival time; (3) cosmesis: type, patient acceptance, surgeon training, and symmetry; and (4) quality of life: general quality of life, breast quality and associated sequelae. No less important, we must consider three additional topics: (5) breast reconstruction; (6) special situations in mastology, where oncoplasty can be used; and (7) training in oncoplasty.

In 2022, given this growing demand, we had the opportunity to serve as the Guest Editorial Board on “*Oncoplastic Surgery for Breast Cancer*” (OSBC) topic. The main focus was to discuss oncoplasty through articles related to the seven main topics previously described. We made a list of the main authors who have been applying oncoplastic techniques and have been publishing it this field. A total of 330 researchers were invited to participate. To be part of this Research Topic, the author must have chosen a Research Topic in the OSBC context and accepted the conditions associated with open access publication. Over one year, 30 researchers considered sending articles, 18 manuscripts were fully submitted, and according to the standards of the journal and the reviewers, 12 articles were accepted within the estimated deadline. Fortunately, we have closed this special volume with the OSBC theme.

This special volume includes 3 reviews and 9 original articles encompassing the seven main themes in oncoplasty. The reviews discuss the multiple aspects associated with breast oncoplasty; the multiple indications, possibilities and results related to the extreme oncoplasty; and methodologies and results for assessing quality of life. In terms of patient selection, there is a publication discussing the impact of nuclear magnetic resonance imaging and another discussing the impact of neoadjuvant chemotherapy and potential therapeutic possibilities. From the point of view of surgical techniques, we have a review related to extreme oncoplasty. We also have original articles showing results associated with the use of dermoglandular advancement flaps and Wise Pattern Mammoplasty. Regarding the evaluation of long-term results, we have articles related to recurrence and conditions associated with unsatisfactory results in BCT. We also have a study related to the use of the latissimus dorsi in breast reconstruction. Under the theme of special conditions, we discuss the use of OSBC in Paget’s disease of the breast. We have one article reporting a successful model of training in oncoplasty. We are happy to organize and assist the discussion of OSBC, allowing breast surgeons to broaden their horizons on such an important topic.

Our patients deserve qualified treatment. All breast surgeons must think about surgical possibilities associated with OSBC and be qualified. Breast oncoplasty is here to stay, and this publication is a gift from the journal, the editors, the reviewers and authors, to breast surgeons and mainly our patients, the reason for this entire publication.

## Author contributions

RV: Conceptualization, Project administration, Supervision, Validation, Writing – original draft, Writing – review & editing. GF: Validation, Visualization, Writing – original draft, Writing – review & editing. DT: Validation, Visualization, Writing – review & editing. CU: Conceptualization, Validation, Visualization, Writing – review & editing. RF-J: Conceptualization, Validation, Visualization, Writing – review & editing.

## References

[1] Curigliano G Burstein HJ Winer EP Gnant M Dubsky P Loibl S. De-escalating and escalating treatments for early-stage breast cancer: the St. Gallen International Expert Consensus Conference on the Primary Therapy of Early Breast Cancer 2017. Ann Oncol (2017) 28:1700–12. doi: 10.1093/annonc/mdx308 PMC6246241 28838210

[2] Audretsch WP Rezai M Kolotas C Zamboglou N Schnabel T Bojar H. Tumor-Specific immediate reconstruction in breast cancer patients. Perspect Plast Surg (1998) 11:71–100. doi: 10.1055/s-2008-1080243

[3] de Andrade Urban C. New classification for oncoplastic procedures in surgical practice. Breast (2008) 17:321–2. doi: 10.1016/j.breast.2007.11.032 18485704

[4] Clough KB Kaufman GJ Nos C Buccimazza I Sarfati IM. Improving breast cancer surgery: a classification and quadrant per quadrant atlas for oncoplastic surgery. Ann Surg Oncol (2010) 17:1375–91. doi: 10.1245/s10434-009-0792-y 20140531

[5] Chatterjee A Gass J Patel K Holmes D Kopkash K Peiris L. A consensus definition and classification system of oncoplastic surgery developed by the american society of breast surgeons. Ann Surg Oncol (2019) 26:3436–44. doi: 10.1245/s10434-019-07345-4 30977016

[6] Patel K Bloom J Nardello S Cohen S Reiland J Chatterjee A. An oncoplastic surgery primer: common indications, techniques, and complications in level 1 and 2 volume displacement oncoplastic surgery. Ann Surg Oncol (2019) 26:3063–70. doi: 10.1245/s10434-019-07592-5 31342388

[7] Silverstein MJ Savalia N Khan S Ryan J. Extreme oncoplasty: breast conservation for patients who need mastectomy. Breast J (2015) 21:52–9. doi: 10.1111/tbj.12356 PMC4303932 25583035

[8] Weber WP Morrow M Boniface J Pusic A Montagna G Kappos EA. Knowledge gaps in oncoplastic breast surgery. Lancet Oncol (2020) 21:e375–e85. doi: 10.1016/S1470-2045(20)30084-X 32758475

[9] Oliveira-Junior I Haikel RL Vieira RAC. Breast-conserving treatment in oncoplastic times: indications, cosmesis, and quality of life. Mastology (2021) 31:e20200040. doi: 10.29289/2594539420200040

